# Modified Radiological Union Score of Tibia (RUST) Scores in Diaphyseal Fractures Treated by the Ilizarov Frame: A Retrospective Analysis Evaluating Reliability

**DOI:** 10.7759/cureus.63533

**Published:** 2024-06-30

**Authors:** Rajani Thakur, Tarani Chetana Naga Sai, Sundeep Kund Reddy Aluka, Tarannum S Sadiq, Bhargavi Paladi, Parineetha Akkala

**Affiliations:** 1 Radiology and Imageology, Nizam's Institute of Medical Sciences, Hyderabad, IND; 2 Orthopaedics, Nizam's Institute of Medical Sciences, Hyderabad, IND

**Keywords:** m-rust score intra observer variability, reliability of m-rust score, m-rust score in tibia with ilizarov frame, m-rust score, rust score

## Abstract

Introduction

The number of cases of tibia diaphyseal fractures treated by Ilizarov fixation is increasing. Fractures with infective etiology and altered bone biology due to the requirement of revision surgery or open wounds, which are often treated by the Ilizarov method, have challenges in ascertaining radiological signs of union. In this study, we aim to demonstrate the application of the modified Radiological Union Score of Tibia (m-RUST) scores in the assessment of fracture union in patients operated by the Ilizarov method. The secondary aim is to assess the interobserver and intraobserver variability of the m-RUST score validated by orthopaedicians and radiologists.

Methodology

A total of 119 patients who were treated with an Ilizarov fixator from February 2017 to December 2023 were included in the study. Four observers (two orthopaedicians and two radiologists) independently applied the m-RUST score for the included patients. Clinical data were not disclosed to the observers who worked independently of each other. Intraclass correlation coefficients (ICC) with 95% confidence intervals (CI) were used to measure the reliability of the m-RUST score. Interobserver reliability was measured by examining the scores of four observers from the second assessment, and intra-observer variability was assessed by a repeat evaluation after two weeks following the first assessment.

Results

The m-RUST score of the 119 X-rays analysed ranged from 8 to 16. The mean score in the first assessment was 11.36±3.51, and in the second assessment was 11.42±3.39. The reliability between all the observers was “substantial agreement” (ICC: 0.74, 95% CI). The ICC among the orthopaedicians was 0.77 and that among the radiologists was 0.72.

Conclusion

The m-RUST score has potential in other long bone fractures such as femur or humerus. Assessment of the m-RUST score in the healing of infective sequel and bone grafting conditions has been found effective. The m-RUST score is a dependable score in evaluating union in tibia fractures treated by the Ilizarov frame.

## Introduction

The tibia is the most commonly fractured long bone with an incidence of 17-21 per one lakh population, and road traffic accidents are the most common (62%) cause of these fractures [1]. Reported epidemiological studies showed that 23.5% of them are open fractures, and this is attributed to the lack of muscular covering to the anteromedial aspect of the tibia [2]. Road traffic accidents, particularly direct blows due to being hit by a vehicle, cause significant soft tissue damage apart from the fracture to the tibia [3]. Complications such as chances of infection or non-union in tibia diaphyseal fractures are higher due to a higher incidence of compound fractures and poor blood supply in the lower third area of the leg. In closed fractures of diaphysis of the tibia, intramedullary (IM) nails are the most preferred form of treatment. However, even with IM nails, cases of delayed union and non-union have been reported in about 16.7%, and this has been attributed to either patient or fracture characteristics [4]. The Ilizarov method has been widely applied to infective non-union of tibial diaphyseal fractures. Extended indications for Ilizarov frame application are poor soft tissue condition of the leg or supra malleolar fractures of the tibia. Upon fracture union, frame removal is necessary, and there are no standardized radiological criteria apart from the presence of well-healed cortices with no fracture line. However, in a revision surgery or infective sequel where the Ilizarov frame is applied, clinical signs of union, absence of tenderness or pain at the fracture site, and ability to full weight bearing without pain appear much earlier than solid radiological parameters of union. This often makes it cumbersome to retain the frame to the patient for a longer duration. Dynamization of the frame by loosening the connecting rods across the fracture and testing the pain on full weight bearing is the only criterion to remove frames in such scenarios.

Whelan et al. proposed the Radiological Union Score of Tibia (RUST) to address the problem of objective criteria for the assessment of radiological union [5]. RUST is based on a number of cortices bridged by the callus and the visibility of the fracture line. It is a novel tool that has higher intra- and interobserver agreement and can delineate different degrees of union proposed for the evaluation of tibial diaphyseal fractures with IM nails. In patients with Ilizarov frame, the fracture site is mostly obscured by a cumbersome apparatus, making it more complex to analyze adding to the altered biology of the bone in most of the cases. Most of the time, complete remodelling with loss of visible fracture occurs late, showing the potential to subdivide the cortical assessment with bridging callus. Hence, the modified RUST (m-RUST) score proposed by Litrenta et al. in 2015 for metadiaphyseal fractures treated with plates or IM nails incorporates the fourth category to the existing RUST score, wherein fracture with callus is further assessed if it is just present or bridging to describe the radiological process to union [6]. In this study, we aim to demonstrate the application of the m-RUST score in the assessment of fracture union in patients operated by the Ilizarov method. The secondary aim is to assess the interobserver and intraobserver variation of the m-RUST score validated by orthopaedicians and radiologists.

## Materials and methods

**Figure 1:**
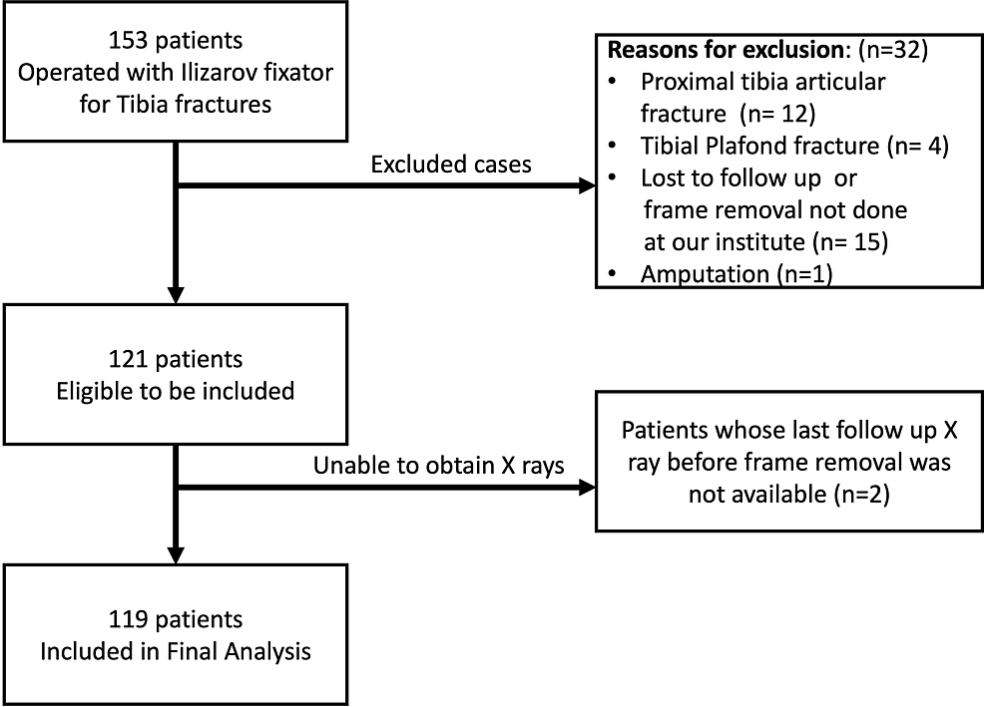
Flowchart demonstrating the enrollment of study participants.

A retrospective study of 153 patients who were operated on for tibia fractures treated by Ilizarov frame at our institute from February 2017 to December 2023 was included in the study. The exclusion criteria were 1) proximal tibia articular fractures, 2) tibia pilon fracture extension, and 3) patients whose frame removal was not done at our institute or lost to follow-up. Of the 153, 34 patients were excluded, leaving a total of 119 patients whose AP (anteroposterior) and lateral view X-rays of the operated leg were available for analysis (Figure 1).

The primary author (RT), who is not an observer in the analysis of X-rays, has collected all the X-rays of the patients before the frame removal from the archive and compiled them for analysis by masking any details of the patient available on the X-ray. Four observers (two radiologists and two orthopaedicians) independent of each other assessed each X-ray with the RUST score. The orthopaedicians who analyzed were not part of the operating team and did not have any access to clinical details such as the duration of the postoperative period, the status of frame removal, and any other details related to the patient. All the DICOM (Digital Imaging and Communications in Medicine) images were exported to a .png (Portable Network Graphics) format and are viewed on a high-definition screen by the observers (iMac, Apple Inc., Cupertino, CA; 27” 5K Retina display). After analysing AP and lateral views, the observer enters the cortex-wise score on a separate Excel worksheet (Microsoft Corporation, Redmond, WA) so that the identity of the observer is not disclosed. Interobserver reliability was measured by examining the scores of four observers from the second assessment, and intraobserver variability was assessed by a repeat evaluation two weeks following the first assessment. After two weeks, the observers are instructed to reassess the X-rays that were reordered in the folder using a program code written in Python programming language [7]. All four observer findings are then analyzed for interobserver and intraobserver variation of m-RUST scores using intraclass correlation coefficients (ICC) with 95% confidence intervals (CI).

**Figure 2:**
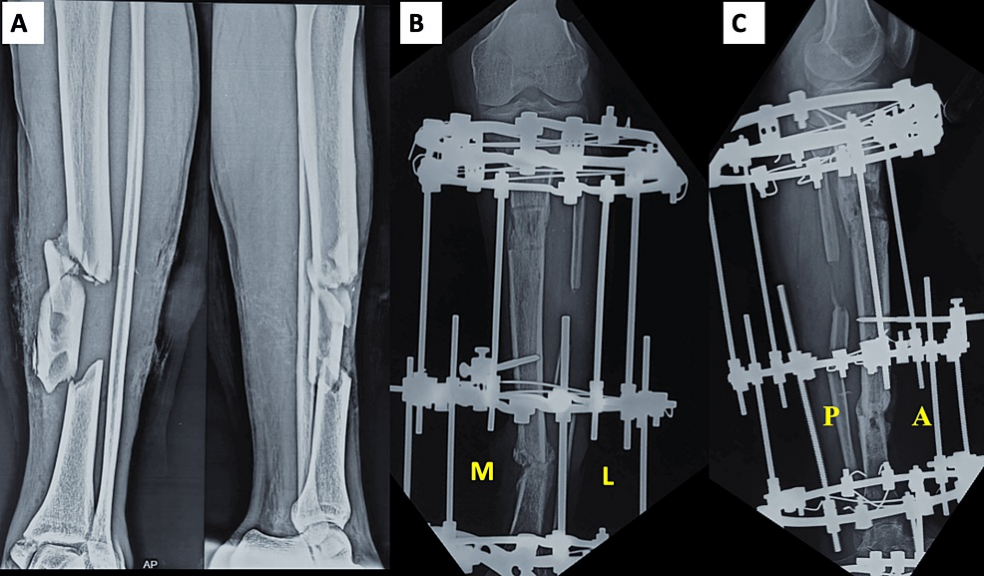
Application of the m-RUST score in the tibia diaphysis fracture treated by the Ilizarov frame fixator. A) Pre-operative X-ray of a segmental tibia fracture that was treated with excision of the infected segmental fragment and Ilizarov fixator application with bone transport. B) AP view of the leg with the medial cortex having no callus (score = 1), lateral cortex having bridging cortex (score = 3). C) Lateral view of the leg with the anterior cortex remodelled with no visible callus (score = 4) and posterior cortex showing bridging callus (score = 3). The total m-RUST score of 11. AP = Anterio posterior; X-ray = Radiograph; m-RUST = Modified Radiological Union Score of Tibia; AP = Anterioposterior; M = Medial; L = Lateral; A = Anterior; P = Posterior

M-RUST score [6]: Each cortex is scored 1-4: 1 = no callus, 2= callus present, 3 = bridging callus, and 4= remodelled with no visible fracture. The total score ranges from 4 to 16 for all four cortices (Figure 2).

Statistical analysis

The scores of all observers were entered in an MS Excel worksheet. ICC values and 95% CI were calculated using Statistical Product and Service Solutions (SPSS, version 20.0; IBM Corp., Armonk, NY). A mean rating, absolute agreement, and two-way mixed effects model were used for analysis. The ICC, used to quantify agreement for a continuous variable, is equivalent to the quadratically weighted kappa (k) for categorical data. A kappa of more than 0.80 is considered “perfect agreement,” a value from 0.61 to 0.80 is considered “substantial agreement” a value from 041 to 0.60 is considered “moderate agreement,” a value from 0.21 to 0.40 is considered “fair agreement” and a value less than 0.20 is considered “slight agreement” [8].

## Results

Of the 119 patients, 39 (32.8%) were female, and 80 (67.2%) were male with an average age of 41.02±18.23 years. Demographic characteristics and patient characteristics are presented in Table 1. All of the included patients had a union, and removal of the frame was done, except for one patient who had amputation following multiple surgeries and was excluded from the analysis.

**Table 1:** Patient characteristics of the study group. RTA – road traffic accident; BMI – body mass index; IM nail – intramedullary nail; % – percentage. The values are given as mean ± SD.

Patient Characteristics	
Age	41.02±18.23
Mechanism of Injury (n)	
RTA – motor vehicle crash	18 (15.1%)
RTA – motorcycle crash	46 (38.6%)
Pedestrian stuck by automobile	42 (35.25%)
Fall	13 (10.9%)
Any comorbidity, n (%)	48 (40.3%)
Smoker, n (%)	22 (18.4%)
Alcohol, n (%)	36 (30.2%)
BMI in kg/m^2 ^, n (%)	
<18	18 (15.1%)
18-25	69 (66.4%)
25-30	22 (18.4%)
>30	10 (8.4%)
Time to surgery from injury date	43.3 ± 21.9
Revision surgery, n (%)	83 (69.7%)
Stage 2 after External fixator removal	45 (54.2%)
IM nail removal and Ilizarov	32 (38.5%
Plate removal and Ilizarov	6 (7.2%)
Bone grafting(autologous cancellous, Iliac crest, n (%)	10 (8.4%)

**Figure 3:**
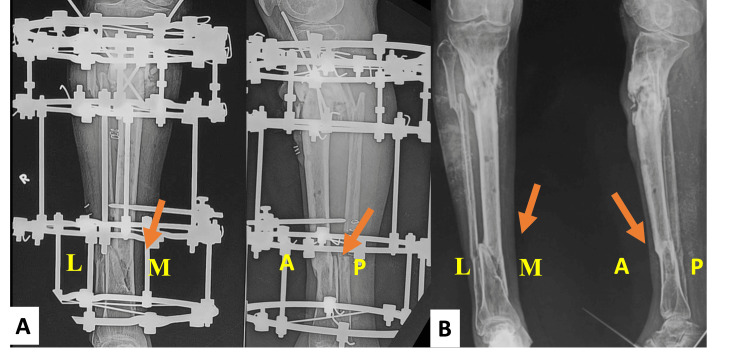
A) Radiograph of the AP and lateral view taken before frame removal of the segmental tibia fracture with upper metadiaphyseal fracture and lower diaphyseal fracture of the tibia treated by Ilizarov frame. Region of interest: Marked in orange arrow - lower third tibia diaphyseal fracture. m-RUST score of medial = 3; lateral = 3; anterior = 4, and posterior = 4. The total m-RUST score assessed is 14. B) AP and lateral view radiographs of the leg showing healed fracture of lower third tibia diaphyseal fracture (marked with an orange arrow) for reference calculation of the m-RUST score. AP - anterio-posterior; M - medial; L - lateral; A - anterior; P - posterior

The m-RUST score of the 119 X-rays analysed ranged from 8 to 16. The mean score after the first assessment was 11.36±3.51 and after the second assessment was 11.42±3.39. The reliability between all the observers was “substantial agreement” (ICC: 0.74, 95% CI). The ICC among orthopaedicians was (0.77) and radiologists (0.72). The ICC of the orthopaedicians and radiologists is comparable (Table 2, Figure 3).

**Table 2:** Intraobserver interclass coefficient (ICC) values for every observer.

Observer	Intraobserver ICC (95% CI)
Radiologists	
Radiologist A	0.72 (0.71-0.73)
Radiologist B	0.73 (0.71-0.75)
Orthopaedicians	
Orthopaedician A	0.78 (0.76-0.79)
Orthopaedician B	0.76 (0.74-0.78)

## Discussion

This study analyses the interobserver and intraobserver variation of m-RUST scores observed by the orthopaedicians and radiologists for tibial diaphyseal injuries treated with the Ilizarov frame fixator. To our knowledge, this is the first study undertaken to analyse the union of tibia fractures by the m-RUST scoring system.

In 1985, Hammer proposed Hammer's index scale, which is based on the presence or absence of fracture line and maturity of bone callus [9]. In 2002, Whelan et al. [10] worked on the inter- and intraobserver variation, and this led them to propose the RUST score in 2010. The RUST score, proposed by Whelan et al. [5], is for tibia fractures treated with IM nailing relying upon the visibility of the callus and is justified by the secondary healing promoted due to the relative stability principle of IM nails. Salih et al. reported a “callus fracture sign,” where in fracture line is visible beyond the cortex through the bridging callus, which is a predictor for hypertrophic nonunion [11].

The concept of m-RUST was developed by Litrenta et al. [6] to study meta-diaphyseal fracture healing with other implants such as LCP plates and IM nails. Owing to more number of complications (nonunion either aseptic or infective), hardware removal followed by the Masquelet technique [12] or Iliarov frame application [13] is widely done for tibial diaphyseal fractures. Often, infections cause alteration in local bone biology, and conventional radiological signs of healing may not be observed in such cases, while clinical signs of healing are evident. This retrospective review was done on 119 patients whose Ilizarov frame was removed following clinical signs of healing and a satisfactory radiological sign of healing as per the treating surgeon's individual decision. With a retrospective analysis of the m-RUST score as radiological parameters of union, the study intended to develop the reliability of the score in tibia fractures treated with the Ilizarov frame. m-RUST has, undoubtedly, good intra- and interobserver reliability even when applied on X-rays with an obscuring Ilizarov frame apparatus. However, the reliability could have been improved by providing a series of follow-up X-rays for comparison by observers.

There are several limitations to this study. As discussed by Whelan et al., the intra- and interobserver reliability of the RUST score analyses only the precision of the score but not the accuracy. However, the results in our study suggest union as observed by the observers in X-rays of patients taken before the dynamization of the Ilizarov frame. The second limitation is the experience level of the observers and very few observers involved in the analysis. Thirdly, we did not evaluate the m-RUST score at regular intervals of follow-up. Applying it on regular follow-up X-rays would have given additional information on the progression of fracture healing over time. Despite this limitation, we believe that the m-RUST score may be used as a supplementary tool to assess fracture healing in patients with the Ilizarov frame fixator. The fourth limitation of our study is not comparing the immediate post-operative X-ray with the X-ray assessed. Most of the time, cortical overlap in spiral fractures or overlap of comminuted fragments can appear as the healed cortex, giving a false impression in assessments.

Apart from conventional X-rays, ultrasonography, contrast-enhanced ultrasonography (CEUS) [14], CT scans, and radionuclide imaging [15] are validated in defining fracture healing. Although CEUS is a non-invasive modality and involves no radiation, it is not a cost-effective tool, which is dependent on radiologist expertise [16]. The most convenient method of evaluating is a conventional X-ray coupled with clinical signs of healing. The m-RUST score would be a supplementary adjunct tool in the assessment of fracture healing.

Future research can focus on applying the m-RUST score and ultrasonography as a tool to investigate the healing of tibia fractures treated by the Ilizarov method to overcome all the shortfalls.

## Conclusions

Our work adds to the growing use of m-RUST scores in the assessment of fracture healing. The m-RUST score has potential in other long bone fractures such as the femur or humerus. Assessment of m-RUST in healing of infective sequel and bone grafting conditions has been found effective. The m-RUST score is a dependable score in evaluating union in tibia fractures treated by the Ilizarov frame. It has good reliability and has a substantial agreement between orthopaedicians and radiologists as observed in our study.
